# The σ^B^-Mediated General Stress Response of *Listeria monocytogenes*: Life and Death Decision Making in a Pathogen

**DOI:** 10.3389/fmicb.2020.01505

**Published:** 2020-07-07

**Authors:** Duarte N. Guerreiro, Talia Arcari, Conor P. O’Byrne

**Affiliations:** Bacterial Stress Response Group, Microbiology, School of Natural Sciences, National University of Ireland Galway, Galway, Ireland

**Keywords:** *Listeria monocytogenes*, σ^B^, stress response, virulence, stressosome, signal transduction

## Abstract

Sensing and responding to environmental cues is critical for the adaptability and success of the food-borne bacterial pathogen *Listeria monocytogenes*. A supramolecular multi-protein complex known as the stressosome, which acts as a stress sensing hub, is responsible for orchestrating the activation of a signal transduction pathway resulting in the activation of σ^B^, the sigma factor that controls the general stress response (GSR). When σ^B^ is released from the anti-sigma factor RsbW, a rapid up-regulation of the large σ^B^ regulon, comprised of ≥ 300 genes, ensures that cells respond appropriately to the new environmental conditions. A diversity of stresses including low pH, high osmolarity, and blue light are known to be sensed by the stressosome, resulting in a generalized increase in stress resistance. Appropriate activation of the stressosome and deployment of σ^B^ are critical to fitness as there is a trade-off between growth and stress protection when the GSR is deployed. We review the recent developments in this field and describe an up-to-date model of how this sensory organelle might integrate environmental signals to produce an appropriate activation of the GSR. Some of the outstanding questions and challenges in this fascinating field are also discussed.

## Introduction

The firmicute *Listeria monocytogenes* is a remarkably robust bacterium with a capacity to grow and survive over a wide range of challenging environmental conditions. It is unusual among food-borne pathogens in being able to grow at refrigeration temperatures and it is very tolerant to high salt concentrations, being able to grow in media containing over 1.5 M NaCl. Additionally, it has an effective protective response against low pH, designated the adaptive acid tolerance response, which allows it to survive at pH values as low as 3.0 for extended periods (O’Byrne and Karatzas, 2008; Dorey et al., 2019b). These traits, combined with the almost ubiquitous occurrence of this microorganism, can allow it to persist in the human food-chain and occasionally establish infections in immunocompromised individuals, elderly people and pregnant women (NicAogáin and O’Byrne, 2016). When they arise, infections can be life-threatening, and outbreaks are associated with high mortality rates, typically 20–30% (Lecuit, 2007).

While many factors contribute to the phenotypic robustness of this pathogen the general stress response (GSR) plays a central role (Gandhi and Chikindas, 2007; Hecker et al., 2007; O’Byrne and Karatzas, 2008; Dorey et al., 2019b). This response is characterized by a general reprogramming of cellular transcription mediated by an alternative sigma factor called SigB (σ^B^), first identified in *L. monocytogenes* just over two decades ago (Becker et al., 1998; Wiedmann et al., 1998). Homologs of σ^B^ are found in most Gram-positive bacteria (Hecker et al., 2007).

In this mini-review, we discuss the recent developments in our understanding of how σ^B^ contributes to stress tolerance and how its activity is regulated in response to stress. We explore its contribution to virulence and analyze the resource implications for the cell of deploying the GSR. We highlight some of the key research questions that remain to be answered in this important field.

## σ^B^-Dependent Robustness in *L. monocytogenes*

The robustness of *L. monocytogenes* is modulated in part by σ^B^, an alternative sigma factor responsible for the upregulation of approximately 300 genes in *L. monocytogenes* (Milohanic et al., 2003; Wemekamp-Kamphuis et al., 2004; Chatterjee et al., 2006; Abram et al., 2008a, b; Raengpradub et al., 2008; Ollinger et al., 2009; Toledo-Arana et al., 2009; Oliver et al., 2010; Shin et al., 2010b; Chaturongakul et al., 2011; Palmer et al., 2011; Ribeiro et al., 2014; Liu et al., 2017; Cortes et al., 2020), including several non-coding sRNA (Nielsen et al., 2008; Toledo-Arana et al., 2009). The σ^B^ regulon, which is not the primary focus of this mini-review, has recently been systematically reviewed by Liu et al., 2019. A subset of approximately 60 genes, identified across strains of *L. monocytogenes* belonging to different lineages, constitute the σ^B^ core regulon (Oliver et al., 2010). Genes comprising the σ^B^ regulon are involved in carbohydrate metabolism (Abram et al., 2008b; Tapia et al., 2020), cell envelope modification (Abram, 2007; Tiensuu et al., 2013), pH homeostasis (Cotter et al., 2005; Karatzas et al., 2010, 2012), osmoregulation (Fraser et al., 2003; Cetin et al., 2004; Wemekamp-Kamphuis et al., 2004; Abram et al., 2008a), regulation of amino acids biosynthesis (Marinho et al., 2019), flagellar biosynthesis (Raengpradub et al., 2008; Toledo-Arana et al., 2009), quorum sensing (Marinho et al., 2020), and antibiotic resistance (Begley et al., 2006). These mechanisms under σ^B^ control have been previously reviewed (O’Byrne and Karatzas, 2008; NicAogáin and O’Byrne, 2016; Dorey et al., 2019b; Liu et al., 2019), and they contribute to the survival of *L. monocytogenes* under a broad range of lethal stresses (Cole et al., 1990; Ferreira et al., 2001; Sue et al., 2003; Wemekamp-Kamphuis et al., 2004; Begley et al., 2005, 2006; Giotis et al., 2008; Palmer et al., 2009; Shin et al., 2010a; Dowd et al., 2011; Feehily et al., 2012, 2013, 2014; O’Donoghue et al., 2016; Curtis et al., 2017; Bourke et al., 2019; Williams et al., 2019). Activation of σ^B^ by one stress often triggers cross protection against other types of stress in *L. monocytogenes* (Begley et al., 2002; Bergholz et al., 2012; Pittman et al., 2014), indicating that a large fraction of the σ^B^ regulon is activated simultaneously. However, many σ^B^-dependent genes are differentially expressed under different growth conditions (Toledo-Arana et al., 2009), suggesting the involvement of additional transcriptional regulators to achieve condition-specific gene expression.

## *L. monocytogenes* Stressosome Structure

**FIGURE 1:**
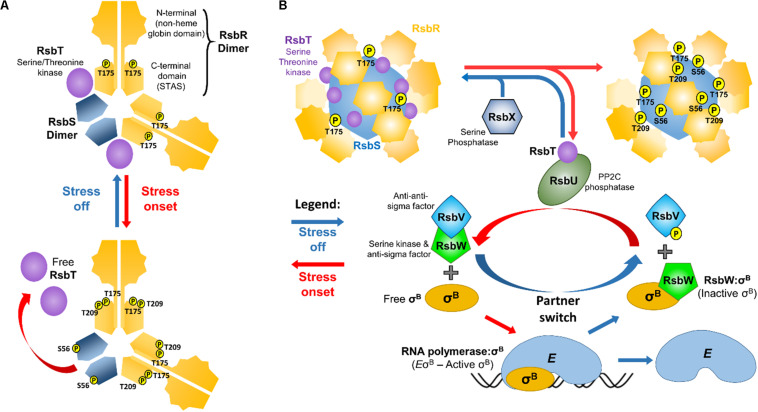
Schematic representation of the o^B^ regulatory pathway in *L. monocytogenes.* **(A)** The repeating hexagonal subunits of the stressosome that are composed of two dimers of RsbR, one dimer of RsbS, and two monomers of RsbT (Williams et al., 2019). It is hypothesized that the N-terminal turrets formed by the RsbR dimers detect stress signals, triggering conformational changes that propagate into the STAS domains, initiating the kinase activity of RsbT. This results in the phosphorylation of RsbR Thr209 and Thr175 and RsbS Ser56, which in turn leads to the release of RsbT from the stressosome. **(B)** Free RsbT interacts with and activates the RsbU phosphatase, which acts on phosphorylated RsbV. Simultaneously, the anti-sigma factor RsbW that binds and prevents σ^B^ from interacting with the RNA polymerase (*E*), releases σ^B^ and preferentially interacts with non-phosphorylated RsbV. σ^B^ can then interact with RNA polymerase forming the holoenzyme *E*σ^B^. Once stress is removed, RsbX dephosphorylates the stressosome (except for RsbR Thr175 which remains phosphorylated; Misra et al., 2011), resulting in the sequestration of RsbT back into the stressosome and inactivating the signal transduction.

To sense environmental changes *L. monocytogenes* relies on a 1.8 MDa supramolecular apparatus designated the stressosome (Figure 1). This stress-sensing organelle is found in members of the proteobacteria, the firmicutes, the actinobacteria, the cyanobacteria, and in the *Bacteroides* and *Deinococcus* groups (Pané-Farré et al., 2005). In *Bacillus subtilis*, the stressosome is composed of RsbRA and its paralogs (RsbRB, RsbRC, RsbRD, and YtvA), RsbS and RsbT forming a pseudo-icosahedral core with turrets on its surface (Chen et al., 2003; Marles-Wright and Lewis, 2008; Martinez et al., 2010; Pané-Farré et al., 2017), the presence of which was later confirmed in *L. monocytogenes*. The *L. monocytogenes* stressosome is composed of RsbR (Lmo0899) and its paralogs RsbR2 (Lmo0161), RsbL (Lmo0799), RsbR3 (Lmo1642), RsbS and RsbT (Impens et al., 2017). The C-terminal domains of RsbS and RsbR fold into Sulfate Transporter and Anti-Sigma (STAS) factor antagonist domains and self-assemble into the stressosome’s core (Aravind and Koonin, 2000). RsbR N-terminal domains, the putative sensory elements of the stressosome, fold into a non-heme globin like structure and associate in dimers (Murray et al., 2005), forming turrets at the complex surface. Pull-down experiments revealed that the RsbR N-terminal domain in *L. monocytogenes* can bind to the small membrane-spanning peptide Prli42, which has been suggested to anchor the stressosome to the cell membrane and to contribute to oxidative stress sensing (Impens et al., 2017). In the same study, the remaining RsbR paralogs were also found associated with the stressosome, the exception being Lmo1842, which was not detected, perhaps consistent with the low transcription levels of the corresponding gene (Wurtzel et al., 2012; Bécavin et al., 2017). In a recent study, *in vitro* assembly of the *L. monocytogenes* stressosome proteins purified from *Escherichia coli*, revealed that it has an icosahedral shape with a 2:1:1 RsbR:RsbS:RsbT stoichiometry and an hexagonal basic structural subunit composed of two dimers of RsbR and one dimer of RsbS (Figure 1A), where the dimeric interfaces form a rigid structure that is responsible for the stressosome integrity (Williams et al., 2019). While the current understanding of the stressosome structure has been thoroughly reviewed in a number of studies (Marles-Wright and Lewis, 2010; Pané-Farré et al., 2017; Tiensuu et al., 2019), there are no structural models available yet that include all RsbR paralogs.

## Insights Into the Mechanism of Stress Sensing by the Stressosome

In *B. subtilis* two residues in the RsbRA STAS domain (Thr171 and Thr205) and one in RsbS (Ser59), can be phosphorylated through the action of the serine/threonine kinase RsbT (Kim et al., 2004), and these residues are all conserved in RsbR and RsbS of *L. monocytogenes* (Ferreira et al., 2004) (Figure 1). In contrast to *B. subtilis*, where all RsbRA paralogs possess phosphorylatable residues, in *L. monocytogenes* only RsbR has these conserved threonines (Thr175 and Thr209). In *B. subtilis* RsbRA Thr171 (*Lm* Thr175) is constitutively phosphorylated (Kim et al., 2004). Indeed, *L. monocytogenes* RsbR Thr175 was also found phosphorylated in the absence of stress, but not *Lm* RsbR Thr209 nor *Lm* RsbS Ser56 (Misra et al., 2011). *B. subtilis* RsbRA Thr205 was found to be phosphorylated only under extreme conditions (Eymann et al., 2011). *Bs* RsbS Ser59 phosphorylation rate seems dependent on *Bs* RsbRA Thr171 and Thr205 phosphorylation (Chen et al., 2004). Amino acid substitutions of *L. monocytogenes* Thr175 and/or Thr209 to Ala resulted in reduced σ^B^ activity and consequently reduced survival in acidic conditions (He et al., 2019).

Following the release of RsbT and consequent activation of σ^B^, a negative feedback mechanism controlled by the phosphatase RsbX allows the stressosome to be reset to its non-stressed dephosphorylated state, which leads to the recapture of RsbT (Voelker et al., 1997; Chen et al., 2004; Eymann et al., 2011). Deletion of *rsbX* produces a constitutive σ^B^ activation and consequently increased survival in acidic conditions (Xia et al., 2016) and a reduced competitiveness against a WT strain, a consequence of the reduced growth rate associated with increased σ^B^ activity (Guerreiro et al., 2020) (see section “σ^B^ Deployment Is a Double-Edged Sword”).

From the plethora of stresses that result in σ^B^ activation, only the blue-light sensing mechanism is well understood. Light is sensed by the phototropin RsbL in *L. monocytogenes* (Ondrusch and Kreft, 2011; Tiensuu et al., 2013; O’Donoghue et al., 2016; Dorey et al., 2019a) and by YtvA in *B. subtilis* (Gaidenko et al., 2006; Ávila-Pérez et al., 2010), both of which have N-terminal light-oxygen-voltage (LOV) domains that associate with a flavin mononucleotide (FMN) (Losi et al., 2002; Ondrusch and Kreft, 2011). Like other RsbR paralogs, RsbL/YtvA associate in homodimers (Buttani et al., 2007; Möglich and Moffat, 2007; Jurk et al., 2010). After blue-light absorption, the FMN forms a covalent adduct with the Cys56 in *L. monocytogenes* RsbL and Cys62 in *B. subtilis* YtvA (Avila-Pérez et al., 2006; Gaidenko et al., 2006; O’Donoghue et al., 2016). The adduct produces a local structural rearrangement in RsbL, propagating into the stressosome core and activating the signal transduction (Salomon et al., 2001; Crosson and Moffat, 2002). Once blue-light is removed, the covalent adduct decays to its ground state (τ_1__/__2_ = 95 min), resetting the protein to its non-stressed state (Chan et al., 2013). Interestingly, *L. monocytogenes* does not activate σ^B^ when exposed to blue-light at 37°C, suggesting that the bond between FMN and residue Cys56 may not form at this temperature (Dorey et al., 2019a). Indeed Chan et al. reported that FMN:RsbL association is reduced as the temperature increases above 26°C. Presumably the absence of an evolutionary pressure to detect light at 37°C, when the pathogen is most likely within the dark confines of a host, produced this temperature-dependent light sensing phenotype.

It is hypothesized that the N-terminal domains of RsbR and the other paralogs are also responsible for the stress signal integration into the stressosome, however, neither the mechanisms involved nor the stress signals being detected are known at present. In *B. subtilis* nutritional stress is sensed through RsbP and RsbQ and integrated into the signal transduction pathway regulating σ^B^ downstream of the stressosome via RsbV (Vijay et al., 2000). In *L. monocytogenes* homologs of RsbPQ are not present, and nutritional starvation is detected by the stressosome through RsbR instead (Chaturongakul and Boor, 2004; Chaturongakul and Boor, 2006; Martinez et al., 2010).

## Signal Transduction

The stressosome, along with proteins that integrate the signal transduction responsible for σ^B^ regulation, are encoded in the *sigB* operon (*rsbR*, *rsbS*, *rsbT*, *rsbU*, *rsbV*, *rsbW*, *sigB*, and *rsbX*). Once stress is sensed, RsbT is released from the stressosome core and is then free to initiate a signal cascade by associating with RsbU, which in turn directs its phosphatase activity toward phosphorylated RsbV (Yang et al., 1996). The anti-sigma factor RsbW, which binds to σ^B^ and blocks its interaction with RNA polymerase (RNApol), has a higher affinity for the dephosphorylated form of RsbV than for σ^B^. RsbV–RsbW interaction restores the phosphorylated state of RsbV through the kinase activity of RsbW, which in turn promotes the reassociation of RsbW with σ^B^, thereby establishing another negative feedback loop (Yang et al., 1996). Once dissociated from RsbW, σ^B^ interacts with RNApol resulting in the upregulation of the σ^B^ regulon. It has been proposed that the signal transduction cascade in *L. monocytogenes* can be inferred from the well-studied *B. subtilis*, since both species share a high level of conservation (Ferreira et al., 2004). Many studies of σ^B^ regulation in *L. monocytogenes* have confirmed that the signal transduction pathways likely function in a very similar way between these two microorganisms (Chaturongakul and Boor, 2004, 2006; Cosgrave, 2010; Shin et al., 2010a; Utratna et al., 2014; O’Donoghue,, 2016; Guerreiro et al., 2020; Hsu et al., 2020).

## Activation of σ^B^ at the Single-Cell Level

Bacterial populations display random fluctuations in the expression of individual genes, metabolite pools, and macromolecular concentrations that generate heterogeneity within the population (Cai et al., 2006; Levine et al., 2013). These differences can give rise to a “bet hedging” survival strategy, where some cells are better prepared for environmental changes and hence have a higher chance of survival under unfavorable conditions. Emerging single-cell analytical methods are increasingly being used to further investigate how σ^B^ activity is regulated at the single-cell level. After exposing *B. subtilis* to mycophenolic acid (MPA), an inhibitor of GTP synthesis that indirectly triggers energy stress, σ^B^ activation was studied using fluorescent protein reporters and time-lapse microscopy (Locke et al., 2011). A series of stochastic pulses of σ^B^ activity was observed in individual cells, with an increased frequency of pulses observed with increasing MPA concentrations. These observations could be explained by fluctuations (noise) in the concentration of some of the key components of σ^B^ regulatory circuit. A minimal mathematical model of the circuit, where fluctuations in the RsbQP phosphatase/RsbW kinase ratio cause sudden increases in σ^B^ activation, exhibited a similar behavior to the experimental observations (Locke et al., 2011). Surprisingly, when a microfluidic-based strategy was used to study σ^B^ activation, the results obtained were somewhat different from those obtained by Locke et al. (Cabeen et al., 2017). In this case, the amplitude of the response increased with the magnitude of the stress, but the frequency of σ^B^ activation remained unchanged (no stochastic pulses were observed). When bacteria were exposed to environmental stresses (osmotic stress and ethanol), a single pulse of activation of σ^B^ was observed, whose amplitude depended on the rate at which the stress increased (Young et al., 2013) or its magnitude (Cabeen et al., 2017). However, strains producing only one of the four RsbR paralogs present in *B. subtilis* displayed repeated stress-activation peaks in single cells, resembling the stochastic activation of σ^B^ reported previously (Cabeen et al., 2017). Pulsing activity of σ^B^ has also been observed during biofilm development, allowing mutually exclusive cell states to co-exist in the same regions of the biofilm and enabling the formation of simple spatial patterns (Nadezhdin et al., 2020). The presence of positive and negative feedback loops within the σ^B^ activation pathway contributes to the generation of noise, with a positive feedback loop amplifying the fluctuations and negative feedback loop, once RsbW is activated, that terminates the pulsing (Nadezhdin et al., 2020). Differences in the experimental approach might affect σ^B^ dynamics differently, causing distinct responses. Future studies will probably need to refine the mathematical models used to predict the activation patterns of σ^B^ in order to resolve the observed experimental discrepancies.

In *L. monocytogenes* heterogeneous activation of σ^B^ was observed when cells were subjected to osmotic shock, with an increased proportion of cells having an active σ^B^ as the magnitude of the stress was increased (Utratna et al., 2012). A similar stochastic behavior of σ^B^ was also observed in another study under similar stress conditions (Guldimann et al., 2017), further supporting the idea of a bet-hedging survival strategy in *L. monocytogenes*.

## σ^B^-Dependent Stress Resistance Role in Virulence

To establish an infection *L. monocytogenes* needs to survive under the harsh conditions of the gastrointestinal (GI) tract, including the acidic conditions of the stomach, osmotic stress in the small intestine, and the presence of bile salts in the duodenum (Sleator et al., 2009; Gaballa et al., 2019; Tiensuu et al., 2019). Survival in the presence of these stresses is partially dependent on σ^B^, as an intragastrically inoculated Δ*sigB* strain exhibits attenuated virulence (Garner et al., 2006; Oliver et al., 2010). σ^B^ regulates the glutamate decarboxylase (GAD) system (Wemekamp-Kamphuis et al., 2002; Cotter et al., 2001a, b, 2005), bile resistance genes such as *bilE* (Fraser et al., 2003; Sleator et al., 2005), *bsh* (Sue et al., 2003; Zhang et al., 2011), *pva* (Begley et al., 2005), and also controls *opuC*, *gbu*, and *betL* to help the bacteria cope with osmotic stress (Fraser et al., 2003; Sue et al., 2003; Cetin et al., 2004; Raengpradub et al., 2008).

A growing body of evidence points toward a complex two-way regulatory network between σ^B^ and the master regulator of virulence, PrfA (Gaballa et al., 2019; Tiensuu et al., 2019). σ^B^ is also responsible for the regulation of the RNA chaperone Hfq which also plays a role in virulence and osmotic stress resistance (Christiansen et al., 2004). The activity of PrfA is crucial for the expression of genes that are important for pathogenesis, including the genes from the Listeria Pathogenicity Island 1 (LIPI-1) and the *inlAB* loci (de las Heras et al., 2011). One of the three promoters that drive *prfA* transcription, P2, is a σ^B^-dependent promoter (Nadon et al., 2002). Under certain forms of stress, transcription from the P2 promoter is enhanced, demonstrating a role for σ^B^ in *prfA* expression (Kazmierczak et al., 2006). There is also a transcriptional overlap between σ^B^ and PrfA regulons, with a group of genes being under the control of both systems (Milohanic et al., 2003). Significantly, it has been shown that σ^B^ plays a crucial role in limiting the availability of branched chain amino acids (BCAA) in *L. monocytogenes*, raising the possibility that σ^B^ might influence PrfA activity via CodY, a global transcription regulator and sensor of BCAA (Marinho et al., 2019). When BCAA availability is low, as they are inside the mammalian host cell, CodY plays a direct role in the transcriptional activation of *prfA* (Lobel et al., 2015). A genome wide analysis of the CodY regulon identified *sigB* as one of the genes that is also directly regulated by CodY, indicating that CodY may promote *prfA* transcription by at least two different mechanisms: directly via binding to the *prfA* gene and indirectly by relieving *sigB* repression (Lobel and Herskovits, 2016). However, *in vitro* binding of CodY to the 5’ coding region of *prfA* is very weak, suggesting that other indirect mechanisms are likely to be involved in CodY-mediated *prfA* activation (Biswas et al., 2020).

Unlike most Gram-positive bacteria, *L. monocytogenes* has the ability to synthesize glutathione (GSH) (Gopal et al., 2005), and is also capable of utilizing exogenous GSH (Portman et al., 2017). It has been shown that GSH allosterically activates PrfA, causing a conformational change that increases binding of PrfA to DNA, promoting the transcription of virulence genes accordingly (Reniere et al., 2015; Hall et al., 2016). The expression of GSH reductase (*lmo1433*), an enzyme that contributes to oxidative stress resistance by reducing GSH disulfide to GSH, is positively regulated by σ^B^ (Kazmierczak et al., 2003). These observations could imply that σ^B^ can indirectly contribute to PrfA activation by maintaining the intracellular GSH levels high through the expression of GSH reductase. This multi-layered regulatory network plays a major role in modifying gene expression in response to environmental stress in *L. monocytogenes* and is central to this pathogen’s remarkable adaptive capacity.

## σ^B^ Deployment Is a Double-Edged Sword

**FIGURE 2:**
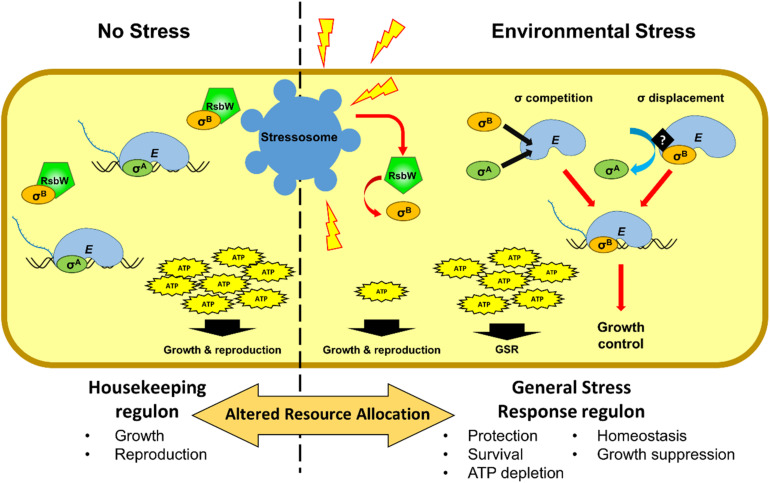
Schematic representation of the alterations in resource allocation that occur during the GSR. Cell growth largely depends on the housekeeping sigma factor σ^A^ in the absence nutrient limitations or stressful conditions. Under these conditions (no stress), most of the transcriptional machinery is dedicated to the transcription of housekeeping genes that preceded by σ^A^ promoters. In the absence of stress, σ^B^ is sequestered by the anti-sigma factor RsbW. At the onset of stress σ^B^ is released from its anti-sigma factor RsbW, resulting in competition between σ^B^ and σ^A^ and the displacement of σ^A^ from a proportion of the RNA polymerase pool. It is possible that the interaction of σ^B^ with RNA polymerase is specifically regulated as has been described in other species. Consequently, genes under σ^A^ control that are associated with growth functions are downregulated and σ^B^ dependent genes (the GSR regulon) are upregulated. The energy resources needed to maintain the general stress response reduces the availability of ATP for growth and reproduction. σ^B^ may specifically regulate growth rate to allow for improved maintenance and repair, thereby increasing the likelihood of survival.

In addition to conferring stress resistance, the activation of σ^B^ also results in reduced growth in *L. monocytogenes* (Figure 2) (Brøndsted et al., 2003; Chaturongakul and Boor, 2004; Abram, 2007; Cosgrave, 2010; Zhang et al., 2013; O’Donoghue et al., 2016; Curtis et al., 2017; Marinho et al., 2019; Sæbø et al., 2019; Guerreiro et al., 2020). It has been hypothesized that living organisms often limit their growth in exchange for increased survival, when conditions are unfavorable due to nutrient limitation (Nyström, 2004). Recently, we have shown that *L. monocytogenes* σ^B^-defective strains exhibit a decreased acid tolerance but have increased growth rates and a competitiveness advantage under mild heat stress (Guerreiro et al., 2020). This growth advantage allows strains with reduced σ^B^ activity to overtake the WT in mixed strain competition experiments. The reason for this growth advantage is not clear at present but three hypotheses seem worth considering. First, competition of different sigma factors for the same allosteric site of the RNApol to produce an active holoenzyme (*E*σ) could redirect transcription away from growth-related functions (Figure 2). In *L. monocytogenes* the availability of σ^B^ to form *E*σ^B^ is ultimately governed by the signal transduction leading to the release of σ^B^ from RsbW. As more σ^B^ is released from RsbW the competition with other sigma factors increases (Figure 2). This potentially impacts the housekeeping σ^A^, which is responsible for the transcription of growth related genes (Österberg et al., 2011). Indeed mathematical models support this type of competition (Mauri and Klumpp, 2014). Whether σ^B^ has a higher affinity for RNApol than σ^A^ or a displacement mechanism exists, as has been shown for *B. subtilis* σ^E^ and σ^K^ during sporulation (Ju et al., 1999), are still unknown. Second, σ^B^ activation may result in the depletion of energy resources to the extent that it has a negative impact on growth. Indeed, exposure to different types of stress results in the reduction of the ATP pool in several bacteria (Antonietti and Ferrini, 1986; Hecker et al., 1989; Antonietti and Tomaselli, 1991). Additionally, Δ*sigB* mutants exhibit higher intracellular ATP levels compared to a WT strain after the exposure to osmotic stress (Xia et al., 2016). In contrast, an Δ*rsbX* mutant has lower ATP levels, as a result of the over-activation of σ^B^ (Xia et al., 2016). Third, it is conceivable that σ^B^ specifically reduces growth as part of an overall damage mitigation strategy in the face of stress. We recently showed that the σ^B^-dependent sRNA, Rli47, blocks isoleucine biosynthesis in *L. monocytogenes* through a direct interaction with *ilvA* mRNA. This interaction results in restricted growth under conditions where isoleucine is limited and suggests a possible role for σ^B^ in controlling growth under those conditions (Marinho et al., 2019). Further studies will be needed to tease these possibilities out fully but it is already clear that σ^B^ has an important impact on fitness and is likely to be subjected to a strong selective pressure. Indeed this may well explain the complexity of the regulatory system controlling σ^B^ activity; deciding precisely when and to what extent σ^B^ should be deployed is critical to resource allocation in times of stress and this ultimately determines fitness (Figure 2).

## Future Perspectives and Challenges

It is over 20 years since the σ^B^ system has been discovered in *L. monocytogenes* and its role in controlling the GSR and the many stress-related phenotypes associated with loss-of-function have been well described. However, there is still much to learn about how its activity is regulated.

Probably the biggest challenge facing the field, and this is also true in *Bacillus*, is that there is very little understanding of what stress signals are detected and how these signals are integrated into the σ^B^ regulatory pathway via the stressosome. The only exception to this is the mechanism that allows photons of blue light to be detected by the stressosome protein RsbL (Chan et al., 2013; O’Donoghue et al., 2016). It is clear that acid and salt and growth-phase all trigger the activation of σ^B^ (Utratna et al., 2011) but the nature of the stress signal detected in each case is unknown, neither is the sensory mechanism known. It is thought that, like RsbL, the N-terminal domains of RsbR or its paralogs (RsbR2, 3, and 4), which are predicted to form turret-like structures that protrude from the surface of the stressosome, are likely to play an important role in signal integration. Whether multiple distinct signals can be detected (possible by virtue of the distinct N-terminal domains of RsbR and its paralogs), or whether a single generic stress-associated signal is detected is still unknown at present. In the case of oxidative stress, it has been proposed that the membrane-spanning miniprotein Prli42 might transduce signals directly to the stressosome through its interaction with RsbR, but the mechanism involved has not been elucidated (Impens et al., 2017).

Although some structural information is available for the stressosome (Williams et al., 2019), high resolution crystal structures of individual components combined with cryo-electron microscopic images of native stressosomes (as opposed to *in vitro* reconstituted stressosomes) will be required to build a clear picture of what the *in vivo* structure of the stressosome is like. Information on subcellular localization and assembly dynamics will also be useful to build a model of where in the cell stress sensing occurs and whether stressosomes are structurally homogeneous *in vivo* or whether different stoichiometries can produce functional differences between them. Single-cell time-resolved approaches will be necessary to see whether structural or stoichiometric differences in stressosomes between cells might contribute to heterogeneity in σ^B^ activity observed within populations subjected to stress. The extent to which individual *L. monocytogenes* cells engage in bet-hedging in response to stressful environmental conditions remains to be fully explored.

Finally the role of the GSR in modulating the virulence of *L. monocytogenes* is still an open question. There are multiple lines of evidence suggesting regulatory crosstalk between σ^B^ and PrfA and these need to be explored further (Gaballa et al., 2019; Tiensuu et al., 2019). While σ^B^ plays an essential role during the GI stage of the infectious cycle, it is less important during the systemic stages, where PrfA appears to be the dominant regulator. Both regulators are modulated by complex multi-layered control circuitry, highlighting the importance to fitness of deploying these systems only when the prevailing conditions are suitable. We have seen clear evidence that there is a significant burden on resources associated with deploying the GSR (Guerreiro et al., 2020) and a similar cost has been reported for inappropriate activation of PrfA (Bruno and Freitag, 2010). Clarification of the nature of the regulatory crosstalk between these systems will give new insights into the biology of this human pathogen as well as suggesting new approaches to control it.

## Author Contributions

All three authors contributed to researching, writing, and editing this mini-review.

## Conflict of Interest

The authors declare that the research was conducted in the absence of any commercial or financial relationships that could be construed as a potential conflict of interest.
